# Fuzzy Self-Tuning Tracking Differentiator for Motion Measurement Sensors and Application in Wide-Bandwidth High-Accuracy Servo Control

**DOI:** 10.3390/s20030948

**Published:** 2020-02-10

**Authors:** Yang Gao, Dapeng Tian, Yutang Wang

**Affiliations:** 1 Key laboratory of Airborne Optical Imaging and Measurement, Changchun Institute of Optics, Fine Mechanics and Physics, Chinese Academy of Sciences, Changchun 130033, China; gaoyang161@mails.ucas.edu.cn (Y.G.); ytwang@ciomp.ac.cn (Y.W.); 2 University of Chinese Academy of Sciences, Beijing 100049, China; 3 Research Institute of Intelligent Control and Systems, Harbin Institute of Technology, Harbin 150001, China

**Keywords:** tracking differentiator, sensor signal processing, fuzzy Self-tuning, motion measurement sensors

## Abstract

Sensor differential signals are widely used in many systems. The tracking differentiator (TD) is an effective method to obtain signal differentials. Differential calculation is noise-sensitive. There is the characteristics of low-pass filter (LPF) in the TD to suppress the noise, but phase lag is introduced. For LPF, fixed filtering parameters cannot achieve both noise suppression and phase compensation lag compensation. We propose a fuzzy self-tuning tracking differentiator (FSTD) capable of adaptively adjusting parameters, which uses the frequency information of the signal to achieve a trade-off between the phase lag and noise suppression capabilities. Based on the frequency information, the parameters of TD are self-tuning by a fuzzy method, which makes self-tuning designs more flexible. Simulations and experiments using motion measurement sensors show that the proposed method has good filtering performance for low-frequency signals and improves tracking ability for high-frequency signals compared to fixed-parameter differentiator.

## 1. Introduction

Signal differential estimation is essential for wide-bandwidth high-accuracy servo controls [1]. High-precision measurement of position, velocity and acceleration signals is a necessary feedback information for various control strategies, including classical PID control [2], sliding mode control [3], and so on. However, due to the limitation of measurement mechanism, some motion measurement sensors, such as velocity sensors and acceleration sensors, have certain measurement noise which limits the accuracy of the measurement. Besides, additional motion measurement sensors are always limited by system installation space and cost [4,5].

In practical engineering, differential signal is extracted from the given input to realize the design of a high order controller. Since pure differential operation is physically impossible, various approximation methods are applied. Classical difference methods obtain the differential signal with the small sampling time. But the signal noise in the original signal will be greatly amplified.

Many researchers have proposed new designs of tracking differentiators to realize signal differential estimations [6,7]. However, for complex signals, fixed parameters in linear tracking differentiator will not only slow down the response speed of the control system, and may even cause system oscillation [8,9].

In recent years, due to its robustness to signal noise and ease of implementation, nonlinear tracking differentiators (NTD) have attracted extensive attention for related researchers. A nonlinear tracking differentiator based on time-optimal control is proposed in [10]. In [11], a finite impulse response filter that discretizes the fractional-order differentiator over functions in Paley-Wiener space is constructed. A high-gain nonlinear tracking differentiator is presented and its weak convergence based on finite-time stable systems is proved [12], but the gains could not be infinite in practice. To cope with the nonlinearities caused by the noise, an improved nonlinear tracking differentiator with hyperbolic tangent function is presented [13]. Nevertheless, a chattering phenomenon occurs in the derivative evaluation due to the existence of discontinuous functions. A high-order continuous nonlinear differentiator with lead compensation is presented in [14]. The high-order nonlinear differentiator obtains the high-order derivatives of a signal, but the chattering phenomenon is reduced. In [15], a new quick-response sliding mode tracking differentiator (new TD) for feedback control of mechatronic systems. It improves response speed and chatter suppression. In [16], a feedforward-constructing NTD was first proposed. It introduces the idea of feedforward in the signal processing algorithm, which provides a new degree of freedom to design. This method makes it possible to reduce the phase lag without changing the filter characteristics. However, in this method, the gain is fixed, which means it is still difficult to obtain optimal performance with wide frequency range of the input signal.

However, some control systems, such as high-speed moving robot systems with a faster dynamic process or servo systems with higher requirements on accuracy and control bandwidth, are more sensitive to the anti-noise ability of differential signals on different frequency and signal phase lag. To further improve the system control performance, it is necessary to study a differential estimation method with better signal adaptability.

Due to above issues, some tracking differentiators with adaptive properties were proposed. A self-adaptive second-order tracking differentiator which can automatically adjust its tracking rate is proposed by [17], where an approximate sigmoid function is designed as the rate switching function. However, due to the change of switching function, the local signal quality cannot be guaranteed. A fourth-order tracking differentiator is introduced to adaptively estimate system states in [18]. In [19], a fuzzy variable parameter is proposed to adjust the parameter of tracking differentiator. In [20], the impact of the algorithm parameter setting on filtering is analyzed in the frequency domain. However, these design processes are too complicated for practical engineering implementations.

Although the NTDs presents excellent tracking performance and anti-disturbance capability, there is always a contradiction between noise resistance for low-frequency signal and tracking performance for high-frequency signals, no matter how the nonlinear function is designed. Due to this reason, it is difficult to guarantee the accuracy of differential estimates in a relatively wide-bandwidth, which leads to the degradation of practical effect of the NTDs, especially in a high-accuracy servo control system.

To solve above problems, it is necessary to design a simple, flexible, and practical method. The method should be able to automatically adjust the parameters according to the input signal frequency. At the same time, switching chattering caused by parameter changes should be suppressed.

In this paper, a fuzzy self-tuning tracking differentiator (FSTD) is proposed for motion measurement sensors, which is able to self-tune the LPF bandwidth of tracking differentiator based on fuzzy inference according to the frequency of the input signal. During differential estimation, the proposed FSTD does not only improve the noise resistance of the low-frequency signal, but also improves signal tracking performance of the high-frequency signal. In brief, the proposed FSTD can effectively conduct differential estimation in a wider bandwidth, and a fuzzy self-tuning design is given. Moreover, we experimentally investigate practical applications of a high-accuracy servo control system, and the results verify the validity of the proposal.

This paper is organized as follows: we briefly discuss problem formulation in Section 2; Detailed design of the proposed fuzzy self-tuning tracking differentiator is introduced in Section 3; Numerical simulation results are given in Section 4; Practical experiments are performed to verify the effectiveness of the proposed approach in a high-accuracy servo control system in Section 5; Results and future perspectives of our work are discussed in Section 6; Finally, the conclusions are presented in Section 7.

## 2. Problem Formulation

Difference calculation is expressed as

(1) $$y\left(t\right)=\frac{1}{h}\left(r_{0}\left(t\right)-r_{0}\left(t-h\right)\right)\approx {\dot{r}}_{0}\left(t\right),$$

where $r_{0}\left(t\right)$ and y(t) refer to the original input signal and the output differential signal, respectively, with *h* being the time constant. Ideally, the original input signal is noise-free, and hence the estimation accuracy of the tracking differentiator depends on time constant *h*. The smaller *h* is, the higher the accuracy of the tracking differentiator would be. However, due to the limitation of the measurement mechanism, high-frequency noises are inevitably mixed into the original sensor signal $r_{0}\left(t\right)$. The differentiator with random noise n(t) is expressed as Equation (2), with $r\left(t\right)=r_{0}\left(t\right)+n\left(t\right)$.

(2) $$y\left(t\right)=\frac{1}{h}\left(r\left(t\right)-r\left(t-h\right)\right)\approx {\dot{r}}_{0}\left(t\right)+\frac{1}{h}\left(n\left(t\right)-n\left(t-h\right)\right).$$

According to Equation (2), the smaller the time constant is, the more serious the signal noise is amplified. When n(t)≡0, Equation (2) is simplified as Equation (1). Let Y(s), R(s) be the frequency representations of y(t), r(t) in the frequency domain, the differential process is rewritten as

(3) Y(s)=sR(s).

In order to improve the anti-noise ability of differential calculation, the differential signal is obtained by using pseudo-differential as Equation (4). LPF is introduced to reduce the influence of noise. For example, the difference module in Simulink uses this method.

(4) $$Y\left(s\right)=\frac{gs}{s+g}R\left(s\right),$$

where *s* is the Laplace operator, $\frac{g}{s+g}$ is a typical first-order LPF, and *g* is the filter parameter and its value is equal to the cutoff frequency.

Further, it is deduced from Equation (4) as Equation (5), where the differential process is viewed as signal $\frac{Y(s)}{s}$ tracks the input signal R(s) under a proportional control. Therefore, this type of differentiator is called a tracking differentiator.

(5) $$\begin{array}{cc} Y(s) & =\frac{gs}{s+g}R\left(s\right)\\ \; & =g\left(1-\frac{g}{s+g}\right)\frac{Y(s)}{s}\\ \; & =g(R\left(s\right)-\frac{Y(s)}{s}). \end{array}$$

For the differential process, with the LPF, in the low-frequency band, the amplitude gain of the LPF is approximately 1, and the anti-noise characteristic of the integral operation is used to obtain an ideal differential value, and there is phase lag at this time.

Obviously, as *g* increases, the estimated value is closer to the true value of noiseless, but as the gain of *g* increases, the gain of noise increases, and the noise reduction capability of the system decreases. As a result, *g* value option needs to balance between noise reduction and true value approximation.

Most researchers focus on how to solve the noise problems, and many algorithms have been proposed. Intuitively, the differentiator structure is split into a combination of differentiation and filtering based on Equation (4). Alternative differentiators are seen as variants of this combination. Whether linear or nonlinear, there are parameters similar to *g*. Researchers have been trying to improve filtering efficiency to reduce the error. Regardless of the filter structure, caused by which the phase lag cannot be ignored. It is increasingly difficult to improve filtering performance based on fixed-parameter design. It thus motivates new ideas on how to make full use of currently existing filters to effectively reduce differentiator design difficulties.

According to above analysis, as the frequency of the input signal increases, the phase lag of the output differential signal also increases. In order to realize the accurate measurement of the input signal in a wide dynamic range, it needs to tune the value of *g* based on the frequency characteristics of the input signal.

## 3. Design of Fuzzy Self-Tuning Tracking Differentiator

As the conclusions analyzed in Section 2, the smaller the value of *g*, the better the noise reduction performance is, and the larger the phase lag is; The larger the value of *g*, the worse the noise reduction performance is, and the smaller the phase lag is.

Therefore, the basic idea of adaptively adjusting the *g* value is obtained: Smaller *g* values are preferable for low-frequency signals, whereas larger *g* values are preferable for high-frequency signals. This relationship is simply described in the form of Equation (6). Value *g* is determined by the signal frequency characteristics and noise. If we describe this relationship in an approximate way, then an adaptation is achieved for *g*. This relationship is either linear or non-linear, depending on the need of system design. In this paper, we describe it using a fuzzy logic system (FLS).

(6) g=F(r,n).

**Figure 1:**
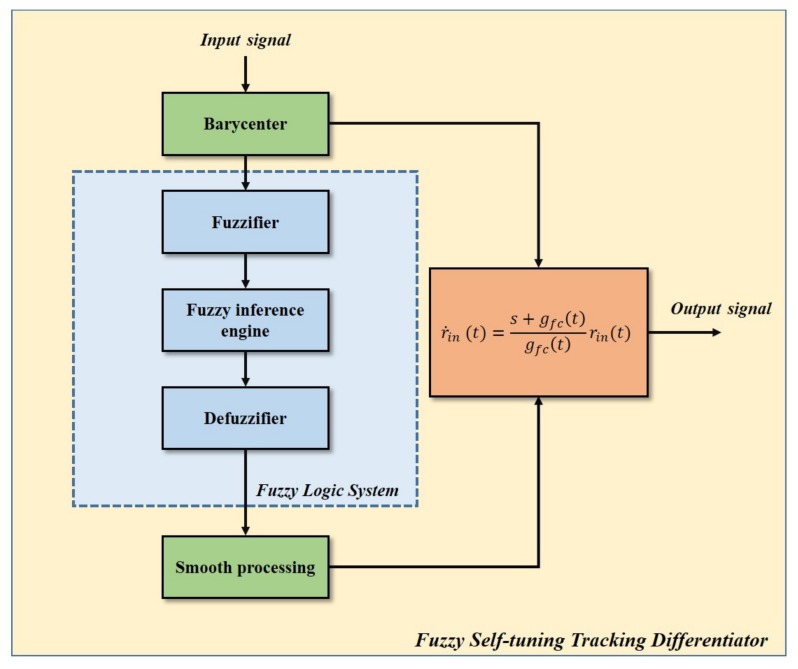
Flow chart of fuzzy self-tuning tracking differentiator.

The flow chart of FSTD is shown in Figure 1. In this section, we will introduce the design of FSTD step by step.

### 3.1. Frequency Barycenter based Signal Frequency Analysis

For general input signals, the information on signals and noise is unknown. As can be seen from above, *g* is related to input signal frequency, so it is necessary to preprocess the input signal to obtain its frequency. For the data obtained by the sensors, it is very difficult to describe all the characteristics in real-time, so the signal information needs to be simplified.

In this paper, we use the frequency barycenter to quantify the frequency information of the input signal. After removing the DC component of the signal, the barycenter of the frequency reflects the center of the signal frequency distribution. For sinusoidal signals, the frequency position is perfectly determined. The calculation of the barycenter is also simple. Experiments show that real-time processing is achieved through a microprocessor, which is practical for experiments. The definition of the frequency barycenter is shown in Equation (7), where $p(f_{n})$ is the frequency spectrum amplitude, $f_{n}$ is frequency, and Ψ is the frequency barycenter.

(7) $$\Psi =\frac{\int p\left(f_{n}\right)f_{n}df_{n}}{\int p\left(f_{n}\right)df_{n}}.$$

For practical discrete systems, data is processed using fast Fourier transform (FFT). Equation (7) is transformed into Equation (8), where *M* is the number of FFT points, $\Delta f_{n}$ is the frequency resolution of the FFT, and P(·) is the result of FFT.

(8) $$\Psi =\frac{\sum_{i=1}^{M/2}P\left(\Delta f_{n}\cdot i\right)\Delta f_{n}\cdot i}{\sum_{i=1}^{M/2}P(\Delta f_{n}\cdot i)}.$$

**Figure 2:**
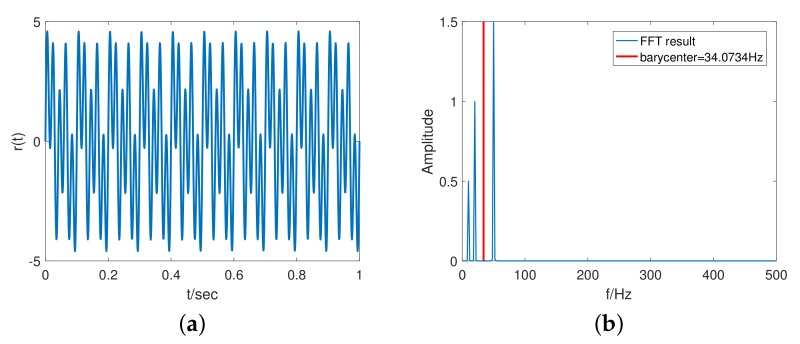
Frequency barycenter calculation: (**a**) Input signal; (**b**) Frequency barycenter analysis result.

Figure 2a shows the signal of Equation (9), where $f_{1}=10Hz$, $f_{2}=20Hz$, $f_{3}=50Hz$, and *n* is a gaussian noise with a mean of 0 and a standard deviation of 0.001. Figure 2b shows the frequency barycenter analysis result.

(9) $$r_{in}\left(t\right)=sin\left(2\pi f_{1}t\right)+2sin\left(2\pi f_{2}t\right)+3sin\left(2\pi f_{3}t\right)+n\left(t\right).$$

By using the frequency barycenter for quantization, the signal characteristics can be described by one variable, so Equation (6) becomes Equation (10).

(10) g=F(Ψ).

### 3.2. Design of Tracking Differentiator Filter Gain Based on Fuzzy Self-Tuning Method

#### 3.2.1. Defining Linguistic Variables and Membership Function

The proposed FSTD consists of a number of input linguistic variables: the frequency barycenter of the input signal Ψ and one output linguistic variable: the cutoff frequency of the LPF, i.e., *g*. The input linguistic variable, the frequency barycenter Ψ of the signal, contains very low (VL), low (L), medium (M), high (H), and very high (VH). Assuming the signal’s bandwidth is bounded, the upper frequency bound is $Fs_{max}$. The input fuzzy set is $[0,Fs_{max}]Hz$. The output language variable, the cutoff frequency *g* of the LPF, contains very small (VS), small (S), medium (M), large (L), very large (VL). The output of the cutoff frequency *g* distribution is $[0,K\cdot Fs_{max}]rad/s,K>1$.

The range selection is usually based on known parameters of the system. For example, in control systems, the bandwidth is used as the input range. To ensure that LPF is effective, $K\cdot Fs_{max}$ does not exceed the sampling frequency of the discrete system. For more complex requirements, it is recommended to flexibly configure based on the prior knowledge of the system.

**Figure 3:**
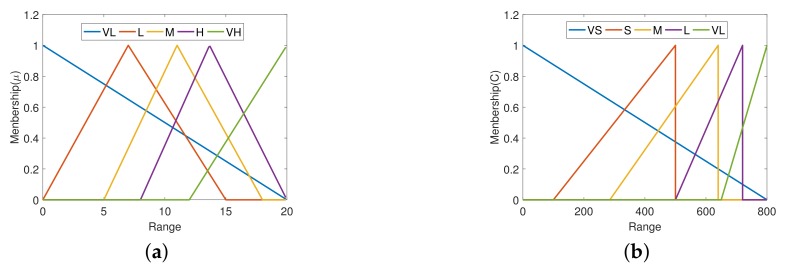
Input and output membership functions: (**a**) Function of the barycenter membership; (**b**) Function of the cutoff frequency membership.

The membership function (MF) is a triangle function. The input membership function (IMF) is shown in Equation (11), and The output membership function (OMF) is shown in Equation (12). The graphical representations of membership functions are shown in Figure 3.

(11) $$\mu_{f_{i}}\left(x\right)=\left\{\begin{array}{cc} \frac{1}{B_{f_{i}}-A_{f_{i}}}\left(x-A_{f_{i}}\right) & x\in [A_{f_{i}},B_{f_{i}}]\\ 1-\frac{1}{C_{f_{i}}-B_{f_{i}}}\left(x-B_{f_{i}}\right) & x\in [B_{f_{i}},C_{f_{i}})\\ 0 & \mathrm{otherwise} \end{array}\right.$$

where $\mu_{f_{i}}\left(x\right)$ is the output of the i-th IMF, *x* is the input of the IMF, and $A_{f_{i}}$, $B_{f_{i}}$, $C_{f_{i}}$ are the vertex coordinates of the IMF, and $0\leq A_{f_{i}}\leq B_{f_{i}}\leq C_{f_{i}}\leq Fs_{max}$. Note that when $A_{f_{i}}=B_{f_{i}}$ or $B_{f_{i}}=C_{f_{i}}$, $\mu_{f_{i}}\left(x\right)=1$.

(12) $$\mu_{c_{j}}\left(y\right)=\left\{\begin{array}{cc} \frac{1}{B_{c_{j}}-A_{c_{j}}}\left(y-A_{c_{j}}\right) & y\in [A_{c_{j}},B_{c_{j}}]\\ 1-\frac{1}{C_{c_{j}}-B_{c_{j}}}\left(y-B_{c_{j}}\right) & y\in [B_{c_{j}},C_{c_{j}})\\ 0 & \mathrm{otherwise} \end{array}\right.$$

where $\mu_{c_{i}}\left(y\right)$ is the output of the i-th OMF, *y* is the input of the OMF, and $A_{c_{j}}$, $B_{c_{j}}$, $C_{c_{j}}$ are the vertex coordinates of the OMF, and $0\leq A_{c_{j}}\leq B_{c_{j}}\leq C_{c_{j}}\leq K\cdot Fs_{max}$. Note that when $A_{c_{j}}=B_{c_{j}}$ or $B_{c_{j}}=C_{c_{j}}$, $\mu_{c_{j}}\left(y\right)=1$.

#### 3.2.2. Defining Fuzzy Rule Base

From the discussion above, we know the relationship between inputs and outputs of the FLS. The knowledge base for the FLS is composed of a series of fuzzy IF-THEN inference rules of the form:

$$R^{i}:\;\mathrm{if}\;x\;\mathrm{is}\;f_{i},\;\mathrm{then}\;y\;\mathrm{is}\;c_{i},\;i=1,2,\ldots ,N.$$

where $f_{i}$ is the i-th input fuzzy set and $c_{i}$ is the i-th output fuzzy set, *N* is the number of fuzzy sets. If N=5, the IF-THEN inference rules are expressed as in Table 1.

#### 3.2.3. Defining Inference and Defuzzification

**Figure 4:**
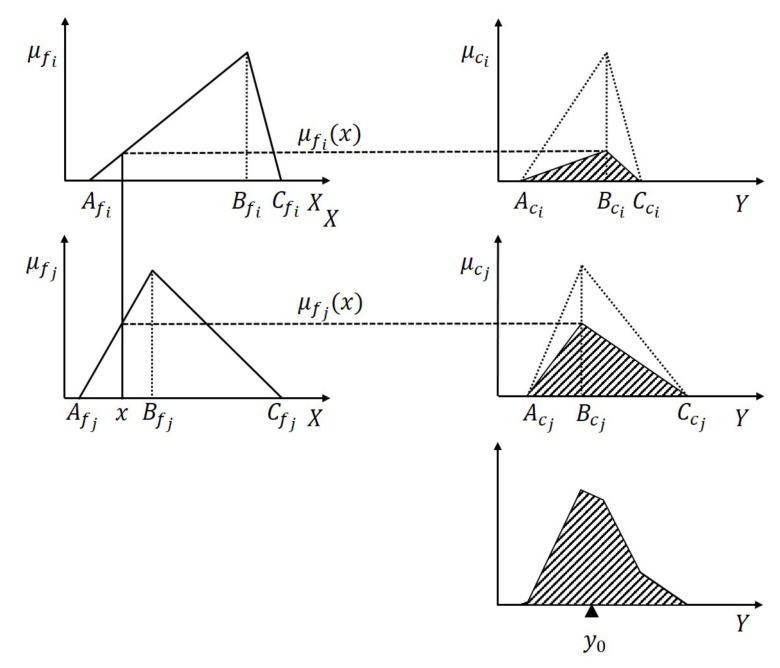
Process of the fuzzy logic system.

The process of the fuzzy logic system is shown in Figure 4. (a) Inference: From the IMF, a truth value $\mu_{f_{i}}\left(x\right)$ is obtained. For each truth value, the OMF $\mu_{c_{i}}$ is scaled by multiplication, and $\mu_{c_{i}^{\prime }}$ is obtained. It is shown as Equation (13). (b) Aggregation: Combine the resulting curves using the SUM operator. The curve sum operation is expressed as $\hat{+}$. It is shown as Equation (14). Where $\mu_{c^{\prime }}$ is the combined curve. (c) Defuzzification: find the center-of-weight of the area under the curve $\mu_{c^{\prime }}$. It is shown as Equation (15). The $y_{0}$ position of this center is then the final output, where $\mu_{c^{\prime }}\left(y\right)$ is the functional expression of curve $\mu_{c^{\prime }}$.

(13) $$\mu_{c_{i}^{\prime }}=\mu_{f_{i}}\left(x\right)\cdot \mu_{c_{i}}$$

(14) $$\mu_{c^{\prime }}=\mu_{c_{1}^{\prime }}\hat{+}\mu_{c_{2}^{\prime }}\hat{+}\ldots \hat{+}\mu_{c_{N}^{\prime }}$$

(15) $$y_{0}=\frac{\int_{0}^{K\cdot Fs_{max}}\mu_{c^{\prime }}\left(y\right)\cdot ydy}{\int_{0}^{K\cdot Fs_{max}}\mu_{c^{\prime }}\left(y\right)dy}$$

In the calculation process, the above curve calculation is simplified according to geometric knowledge. The centroid of the combined shape is directly calculated. The centroid of the combined shape is related to the centroid coordinates and area of sub-shapes. The centroid coordinates $G_{j}$ and area $S_{j}\left(x\right)$ is obtained from Equations (16) and (17). The centroid of the combined shape is obtained from Equation (18).

(16) $$G_{i}=\frac{A_{c_{i}}+B_{c_{i}}+C_{c_{i}}}{3},$$

(17) $$S_{i}\left(x\right)=\frac{\mu_{f_{i}}\left(x\right)\left(C_{c_{i}}-A_{c_{i}}\right)}{2},$$

(18) $$y_{0}=\frac{\sum_{i=1}^{N}S_{i}\left(x\right)\cdot G_{i}}{\sum_{i=1}^{N}S_{i}\left(x\right)}.$$

The form using frequency barycenter Ψ and output parameters of FLS $g_{f}$ can be expressed as

(19) $$g_{f}=\frac{\sum_{i=1}^{N}S_{i}\left(\Psi \right)\cdot G_{i}}{\sum_{i=1}^{N}S_{i}\left(\Psi \right)}.$$

### 3.3. Smooth Processing of Tracking Differentiator Filter Gain

**Figure 5:**
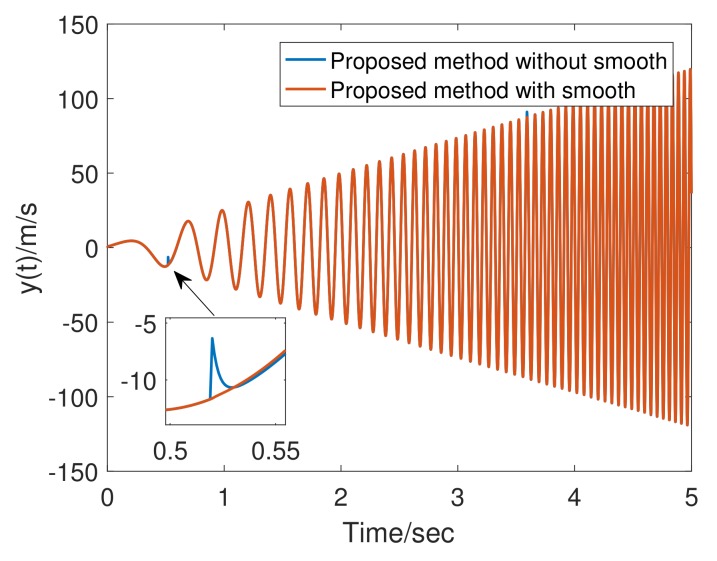
Smooth processing result.

Based on the proposed design method for tracking differentiator parameters in Section 3.2, the $g_{f}$ in FSTD varies with the input signal frequency. Since the FFT requires piecewise calculations, the numerical change of $g_{f}$ is discontinuous with time, leading to the chattering of a differential signal at its switching point. We apply a Sigmoid function as Equation (20) to transform the discontinuous switching into a continuous one.

(20) $$g_{fc}\left(t\right)=\frac{A}{1+e^{-\alpha (g_{f}\left(t\right)-\beta )}},$$

where $g_{fc}\left(t\right)$ refers to smoothed $g_{f}\left(t\right)$, $A=g_{f}\left(t\right)-g_{f}\left(t-1\right)$ is the output amplitude of the function, α affects the width of the smooth zone, and β affects smooth zone center. In Figure 5, both smoothed and unsmoothed tracking differentiator outputs in the simulation are given. We shortly conclude that adding a smoothing link effectively avoid chattering caused by parameter switching.

The construction of the proposed fuzzy self-tuning tracking differentiator is demonstrated in Equation (21), where $y_{FATD}$ is the differential output of $r_{in}$ after FATD and $g_{fc}$ is an adaptive time-varying parameter.

(21) $$y_{FATD}=\frac{g_{fc}}{s+g_{fc}}r_{in}\approx {\dot{r}}_{in}.$$

## 4. Numerical Simulation

To demonstrate the tracking performance of the proposed FSTD, a set of numerical simulations are carried in MATLAB simulation, compared with the traditional tracking differentiator (TTD) as Equation (4) with g= 300 rad/s. The simulation parameter settings are given in Table 2.

**Figure 6:**
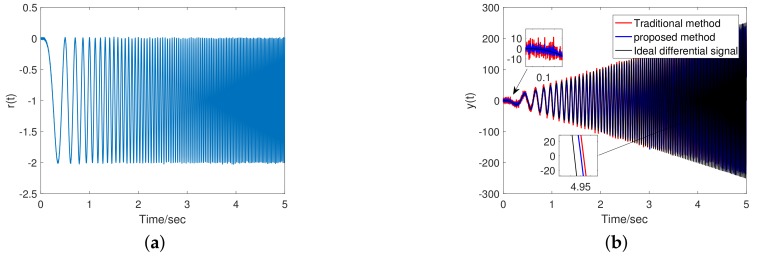
Numerical simulation results: (**a**) Input signal: x(t); (**b**)Output signal: y(t).

In order to verify the self-tuning characteristics of FSTD, we conduct a simulation to use a signal in Equation (22) with changing frequency regarding time, as input to the tracking differentiator. The signal waveform is shown in Figure 6a, and

(22) $$r_{in}\left(t\right)=cos\left(2\pi f\left(t\right)\cdot t\right)-1+n\left(t\right);$$

where f(t)=4t, n(t) is white noise with mean of 0 and variance of 0.001. Differential results of TTD and FSTD are shown in Figure 6b.

**Figure 7:**
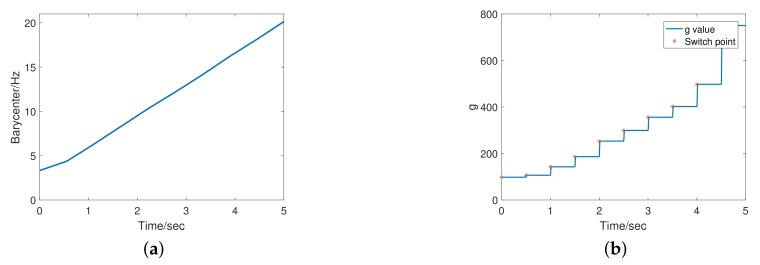
Numerical simulation results: (**a**) Frequency barycenter identification result; (**b**) The *g* value obtained by the proposed method.

According to the simulation results, in the low-frequency region, the FSTD has better filtering performance and the noise amplitude is significantly reduced; in the high-frequency region, the phase lag of the FSTD is smaller. And due to the smoothing process, there is no significant chattering at the switching point. Frequency of input signal was increased from 0 to 20 Hz in 5 s. Figure 7a shows the frequency barycenter change curve calculated by FSTD. This result shows that in the case of a large signal-to-noise ratio, the frequency barycenter is able to describe the frequency characteristics. Figure 7b shows the smoothed *g* value of the FSTD. It demonstrates the expected adaptive effect of FSTD.

## 5. Experiments and Application

**Figure 8:**
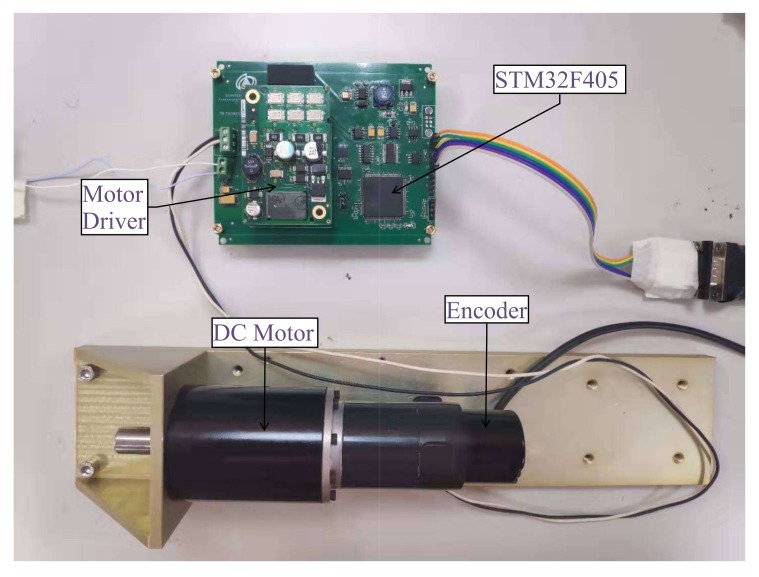
Experimental System.

Practical experiments were implemented on a wide-bandwidth high-accuracy servo control system. We differentiated the signal output by the motion sensor and applied the differential result to the control system. This experiment verifies the performance difference between TTD and FSTD. Figure 8 shows a photograph of the experimental device. The experimental servo control system was composed of a microprocessor system, a DC motor, an incremental optical-electrical encoder, motor drivers, and etc. In the experiment, algorithms were realized by programming an ARM-based (STM32F405) embedded system. All programs were scheduled in C language.

**Figure 9:**
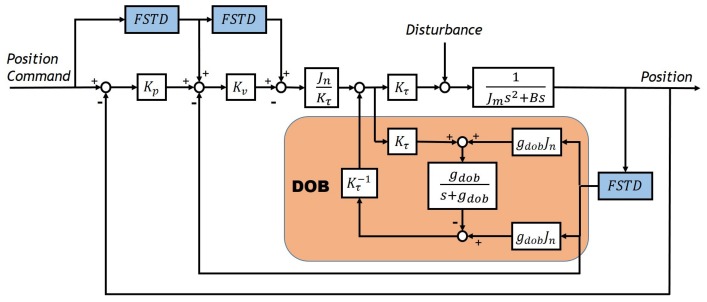
Block diagram of control system.

Figure 9 shows the block diagram of the control system. The control system employs a combination of PD controller, Disturbance observer (DOB), and a feedforward controller. The system is a typical angular position control system. The motion measurement sensor obtains the angle value response, and the control command is the angle value. There are many differentiating operations existing in the system: PD controller needs angle value response and speed value for feedback control; the DOB needs speed value to estimate disturbance [21]; the feedforward controller needs the commanded speed and acceleration to compensate for the phase.

**Figure 10:**
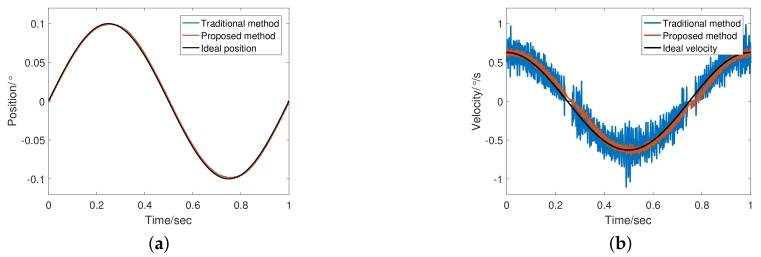
Response Comparison at 1 Hz: (**a**) Comparison of position response at 1 Hz; (**b**) Comparison of velocity estimation at 1 Hz.

**Figure 11:**
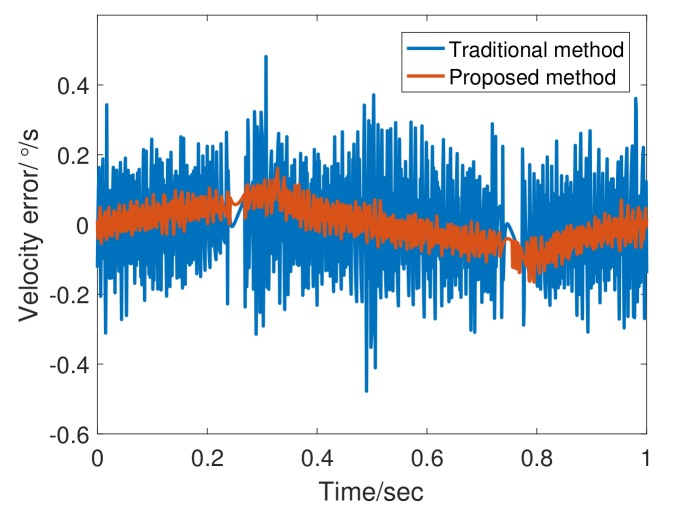
Angular velocity error of TTD and fuzzy self-tuning tracking differentiator (FSTD) at 1 Hz.

**Figure 12:**
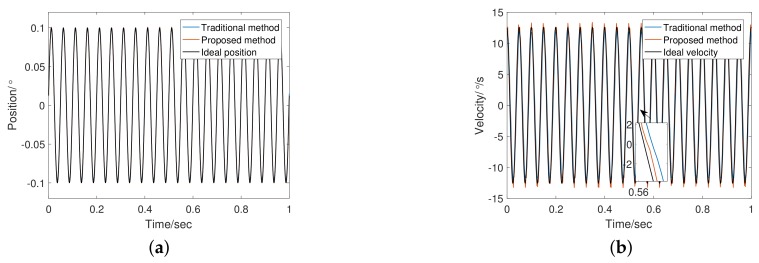
Response Comparison at 20 Hz: (**a**) Comparison of position response at 20 Hz; (**b**) Comparison of velocity estimation at 20 Hz.

**Figure 13:**
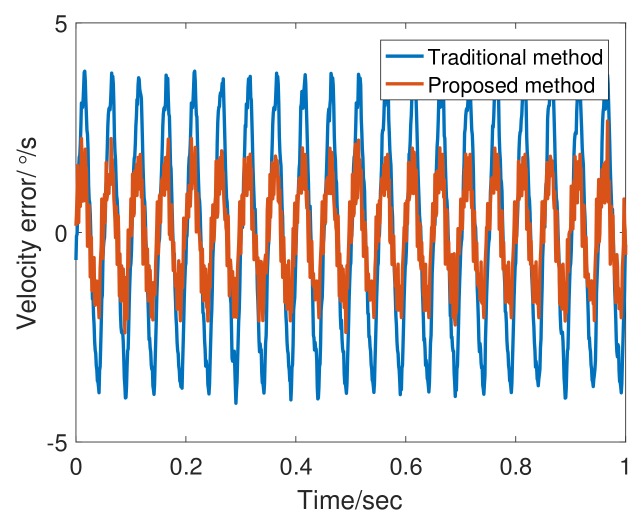
Angular velocity error of TTD and FSTD at 20 Hz.

Table 3 lists the experimental parameters. In addition to the tracking differentiator, the structure and parameters of the system remain unchanged. The experiments compare the effects of tracking differentiator changes on performance. Given input signals of sinusoid command of frequency 1 Hz and 20 Hz to the system, we obtained experimental results as shown in Figure 10, Figure 11, Figure 12 and Figure 13.

Figure 10 shows the encoder output and a differential speed at 1 Hz. The adaptive bandwidth of FSTD is 79.47 rad/s. Obviously, the tracking differentiator shows excellent filtering performance, and the phase lag of the differential is also acceptable due to the presence of feedforward controller. Figure 11 shows that in terms of the angular velocity error, FSTD better than TTD.

Figure 12 shows the encoder output and velocity at 20 Hz. the adaptive bandwidth of FSTD is 748.21 rad/s. The phase lag of FSTD is smaller. From the error curve shown in Figure 13, the phase lag component is greater than the noise component in the 20 Hz signal error. Therefore, compensating for phase lag can more effectively reduce errors. FSTD is better than the traditional method.

## 6. Discussion

From the simulation and experimental results, the self-tuning characteristics of FSTD can effectively solve the shortcomings of tracking differentiator in terms of performance. In particular, it shows excellent adaptability for wide-bandwidth high-accuracy sensor signals. Further, the proposed method is not only for the tracking differentiator in Equation (4), but for all linear or non-linear tracking differentiator systems. In such systems, parameters like *g* would affect the filtering characteristics [22]. These parameter designs need to find a balance between phase lag and noise suppression. The frequency barycenter and the FLS method in FSTD is extended to solve this type of problems.

In addition, for some systems, the tolerance for phase lag is much larger than noise. This paper has also this tendency when designing fuzzy logic systems. But for other special requirements, the design flexibility provided by fuzzy logic systems is sufficient.

## 7. Conclusions

In this paper, a self-tuning differentiator is proposed based on a fuzzy logic system. Currently, there is no low-pass filters capable of both tracking and noise reduction. The self-tuning method effectively expand the range of use of existing filters. By quantifying the characteristics of the signal using the frequency barycenter method, the proposed method achieves adaptive filter bandwidth, widening the range of the usage of tracking differentiators. Simulation and experimental results show that compared with the traditional tracking differentiator, FSTD shows a better filtering ability for low-frequency signals and a better tracking ability for high-frequency signals. The effect of parameter changes on performance is reduced by a smoothing method. By differentiating the angle signal of the photoelectric encoder, the control accuracy of the closed-loop control system is effectively improved under the same parameters, which proves the practicalness of FSTD.

## Figures and Tables

**Table 1:** The fuzzy rule base.

Fuzzy Rules	1	2	3	4	5
(IF) The signal Barycenter Ψ	VL	L	M	H	VH
(THEN) The cutoff frequency *g*	VS	S	M	L	VL

**Table 2:** Parameters in simulation.

Parameters	Symbols	Values
Sampling time	st	0.001 s
FFT length	*M*	512
$g_{fc}$ update time	$t_{fc}$	0.512 s
Smooth parameter	α	100
Smooth parameter	β	t+0.02
Signal frequency range	$Fs_{max}$	20 Hz
LPF bandwidth	$K\cdot Fs_{max}$	800 rad/s

**Table 3:** Parameters in experiments.

Parameters	Symbols	Values
Inertias	$J_{m}$	0.00173 kgm${}^{2}$
Damping Factor	*B*	0.00000126 kg/s
Nominal inertias	$J_{n}$	0.0017 kgm${}^{2}$
Motor thrust coefficient	$K_{\tau }$	5.76 Nm/A
Position control gain	$K_{p}$	50 s${}^{-2}$
Velocity control gain	$K_{v}$	0.12 s${}^{-1}$
DOB cutoff frequency	$g_{dob}$	400 rad/s
TTD bandwidth	$g_{TTD}$	400 rad/s
Signal frequency range	$Fs_{max}$	20 Hz
LPF bandwidth range	$K\cdot Fs_{max}$	800 rad/s
Sampling time	st	0.001 s
FFT length	*M*	512
$g_{fc}$ update time	$t_{fc}$	0.512 s
Smooth parameter	α	100
Smooth parameter	β	t+0.02
